# Ionized alkaline water reduces injury in BALB/c mice infected with *Leishmania amazonensis*

**DOI:** 10.1371/journal.pone.0280695

**Published:** 2023-07-06

**Authors:** Karina Miyuki Retamiro, Karine Campos Nunes, Aline Pinto Zani, Caroline Pinto Zani, Laiza Bergamasco Beltran, Sueli de Oliveira Silva, Francielle Pelegrin Garcia, Tânia Ueda-Nakamura, Rosangela Bergamasco, Celso Vataru Nakamura

**Affiliations:** 1 Laboratory of Technological Innovation in the Development of Pharmaceuticals and Cosmetics, State University of Maringá, Maringá, Brazil; 2 Laboratory of Management, Preservation and Environmental Control, State University of Maringá, Maringá, Brazil; Oregon State University, UNITED STATES

## Abstract

Ionized water has been reported to contribute to the tissue repair process and wound healing. Water purifiers can generate ionized water by means of activated charcoal with silver and minerals, the main purpose of which are to reduce microbiological and physicochemical contaminants. Moreover, when water is subjected to a magnetic field an organization of water molecules occurs due to the presence of mineral salts. The resulting water is thus more alkaline, which has been shown to be non-toxic to mice and can actually prolong survival. Cutaneous leishmaniasis is a neglected tropical disease, caused by obligate uni- and intracellular protozoa belonging to the genus *Leishmania*, that can manifest in the form of skin lesions. Thus, the objective of this study was to compare the evolution of disease in *L*. *amazonensis*-infected BALB/c mice that received tap water (TW) or ionized alkaline water (IAW). As a control, additional groups of mice receiving TW or IAW were also treated with the antileishmanial miltefosine. All mouse groups received either TW or IAW as drinking water 30 days prior to infection and the groups continued to receive the respective drinking water for 4 weeks, after which the blood and plasma were collected. Biochemical assays of aspartate aminotransferase, alanine aminotransferase, gamma-glutamyl transferase, creatinine, urea, glucose, triglycerides, and cholesterol were performed, in addition to hematology tests. There was a significant decrease in the volume of the lesion for groups that received IAW, in which the ingestion of ionized alkaline water favored the non-evolution of the lesion in the footpads of the animals. The results of the blood count and leukogram tests were within the normal values for BALB/c mice demonstrating that ionized water has no toxic effects on blood factors.

## Introduction

Leishmaniasis is caused by obligate uni- and intracellular protozoa of the mononuclear phagocytic system. These protozoa are flagellates of the genus *Leishmania*, belonging to the family *Trypanosomatidae* [1, 2]. Leishmaniasis, despite being a neglected tropical disease, still affects many individuals. The etiological vectors of this disease are insects of the *Psychodida* family of the genus *Lutzomyia* in the Americas, especially in Brazil [3]. There are two main types of the disease, visceral and tegumentary leishmaniasis. The tegumentary type can be divided into mucocutaneous, diffuse, and localized cutaneous leishmaniasis, the latter of which is the most common form [4]. The three main species of *Leishmania* responsible for the tegumentary form of the disease in Brazil are *L*. (*Viannia*) *braziliensis*, the most prevalent species, followed by *L*. *amazonensis*, and *L*. *guyanensis* [5]. The immune response starts right after the bite of an infected vector and depends on several factors such as the recruitment of neutrophils and monocytes to the infection site in the skin. The control of infection depends on the production of interferon (IFN)-γ by CD4^+^ lymphocytes and other cytokines that try to kill the parasites. However, if this response is exacerbated or not adequate, it may lead to progression of the lesion [6, 7].

Water is an essential nutrient for humans since it assists in body homeostasis. The maintenance of a good state of hydration brings several health benefits, promoting improvements in quality of life, as well as preventing chronic diseases and aging [8, 9]. In particular, alkaline water has been demonstrated to have positive effects in the organism that may be associated with its ability to neutralize or even eliminate free radicals present in cells and thus prevent oxidative damage. Furthermore, the consumption of alkaline water favors an acid-base balance, giving a better state of hydration [10, 11]. It has already been reported in the literature that mice treated with alkaline water did not present any toxic effect on the heart, brain, kidneys, liver, and intestine, besides demonstrating a higher survival rate [12].

Some substances contained in the water are of great concern. Chlorine is often used by treatment plants for the elimination of pathogenic microorganisms; however, when free chlorine reacts with other organic compounds such as fulvic and humic acids this generates organic and inorganic chloramines. This by-product has been proven to have undesirable effects on the organism, since its association with cancer is already reported [13, 14].

Water purifiers seek to reduce microbiological and physicochemical contaminants using activated carbon with silver, quartz, and minerals. Through this removal of impurities, alkaline water remains, without the odor and taste of chlorine [15]. Furthermore, magnets may be present in the water purifier, so that when water is subjected to the magnetic field an organization of water molecules occurs due to the presence of mineral salts in water. The presence of infrared aids in the formation of clusters (smaller molecules). According to the World Health Organization (WHO) organized and smaller molecules favor the hydration of cells contributing mainly to the circulatory system. Therefore, the objective of this study was to evaluate the influence of the ionized alkaline water (IAW) in BALB/c mice infected with *L*. *amazonensis*, comparing it with the tap water (TW).

## Material and methods

### Water supplementation

Tap water (TW) was obtained in the laboratory and the pH and chlorine content was adjusted according to the specifications of the Brazilian Ministry of Health (Ordinance N°888 of May 4, 2021) for drinking water. These specifications dictate that the chlorine content should be up to 2.0 mg/mL and the pH should be between 6.0 and 9.5, and thus the TW was prepared to 2.0 mg/mL chlorine and a pH of 6.0.

Ionized alkaline water (IAW) was obtained by passing the previously prepared TW through the Purific^**®**^ filter containing a magnet supplied by the company Água Pura Carbontec Ltda (Maringá, PR, Brazil). The monitoring of pH and chlorine content, as well as the preparation of TW and IAW, was performed three times a week. In addition, a water ionization device was placed inside the water support of mice of all groups.

### Cultivation of promastigote forms of *L*. *amazonensis*

Promastigote forms of *L*. *amazonensis* (WHOM/BR/75/JOSEFA strain) were cultivated as described by Karam et al. (2020) [1] at 25°C in Warren medium (infusion of brain and heart plus hemin and folic acid, pH 7.4) supplemented with 10% inactivated fetal bovine serum (FBS; Gibco Invitrogen, Gaithersburg, MD, USA).

### *In vivo* experimental protocol

This project was approved by the Ethics Committee on Animal Use (CEUA) of the State University of Maringá (UEM), N° 5789051120. Male BALB/c mice, between 4 and 6 weeks of age (20–25 g), were obtained from the UEM Central Laboratory. The animals were housed in the laboratory under the following conditions: the temperature was between 18 and 22°C, the humidity was between 45 and 55%, and the light/dark cycle was 12 h. The animals had feed (Nuvilab^®^) and water *ad libitum*. Mice were weighed throughout the entire experimental period.

**Fig 1:**
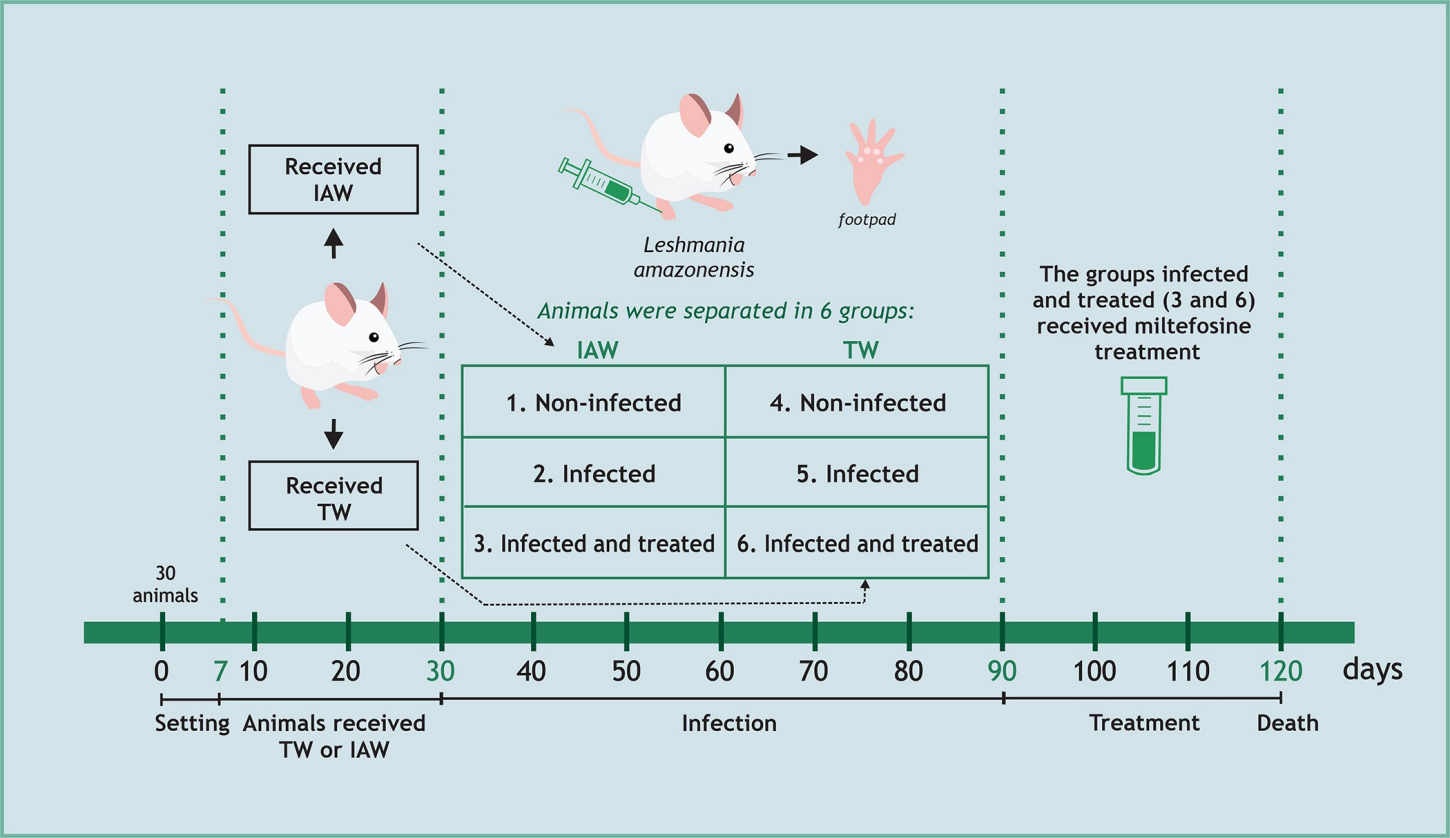
Schematic timeline of the *in vivo* investigation.

Initially, the mice were set for a week after entering the experimental animal house. The mice were then separated into 2 groups of 15 animals each, in which one group received TW and the other IAW as drinking water for 30 days. Then, the groups were further divided, generating 6 groups (5 mice per cage) in total: (1) non-infected and received IAW; (2) infected and received IAW; (3) infected, received IAW and treated with miltefosine; (4) non-infected and received TW; (5) infected and received TW; (6) infected, received TW and treated with miltefosine. After the division of the groups, infection with *L*. *amazonensis* was performed in groups 2, 3, 5, and 6, as described in the next section, and the mice were left for 60 days for the lesion to appear. After this period, miltefosine treatment was performed in groups 3 and 6 for 30 days. After treatment was performed euthanasia, using isoflurane, 2–4%, was administered by inhaled route. After this, post-mortem experiments were performed (Fig 1).

### Infection with *L*. *amazonensis*

Infection with *L*. *amazonensis* was performed as described by Kaplum et al. (2016) [16]. Briefly, four groups (2, 3, 5, and 6) were infected using promastigotes in the stationary phase of growth. With the aid of a 27.5 caliber needle, 20 μL (1 x 10^7^ promastigotes) were subcutaneously inoculated in the left footpad of the mice. The development of the lesion was monitored weekly by measuring the volume of the infected leg with a caliper (DIGIMESS 150 mm). Visual monitoring was also performed to observe the texture of the hair, edema, and redness. For groups 3 and 6, miltefosine was administered daily via gavage, diluted in TW or IAW, for the concentration of 20 mg/kg for 30 days.

### Blood and plasma samples from mice

At the end of the experimental period, blood was withdrawn, EDTA-treated (BD Vacutainer^®^ 2 mL) and centrifuged at 2,000 x *g* for 15 min. Total blood and plasma samples were collected and immediately analyzed or stored at -80°C until analysis. Hematological parameters such as blood count and leukogram were analyzed.

### Biochemical analysis

Biochemical assays for aspartate aminotransferase (AST), alanine aminotransferase (ALT), gamma-glutamyl transferase (GGT), creatinine, urea, glucose, triglyceride, and cholesterol were performed with the plasma of the mice following the manufacturer’s instructions (Laborlab^®^) and readings were performed on spectrophotometer (Microplate Reader Flexstation^®^ 3 Multimode Plate Reader, Molecular Devices).

### Statistical analysis

The experimental data was submitted to statistical tests, using either the one-way or two-way ANOVA test, followed by the Tukey multiple comparison tests, considering p <0.05 as significant. The analyses were performed using the software GraphPad Prism 5 (GraphPad Prism Software, San Diego, CA, USA, 2007).

## Results

### Water purifiers increase the pH and reduce chlorine

Tap water (TW) and ionized alkaline water (IAW) were prepared three times a week, during which chlorine and pH was monitored. The mean values for these parameters were calculated (Table 1), which showed that the pH of the IAW was higher than the TW by 1.25, making the water alkaline. The filter used to produce the IAW was also able to remove 91% of chlorine from the TW.

**Table 1:** Values of pH and chlorine concentration of tap water (TW) and ionized alkaline water (IAW) used in the experiments.

Type of water	pH	Chlorine concentration (mg/mL)
TW	6.68 ± 0.31	1.69 ± 0.13
IAW	7.93 ± 0.20	0.16 ± 0.03

### Weight of mice

**Fig 2:**
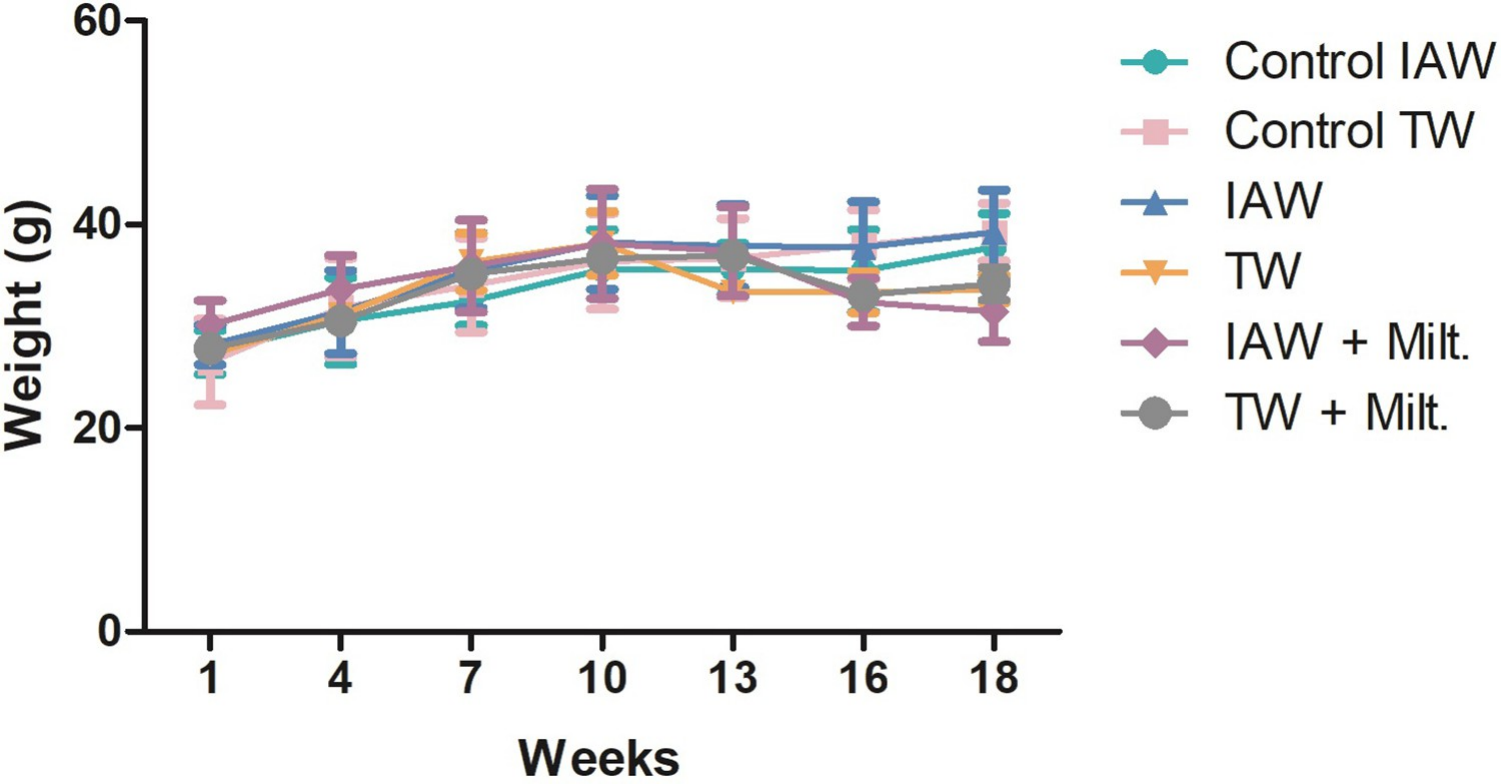
Weight (g) of non-infected control and infected BALB/c mice. Mouse groups received either tap water (TW) or ionized alkaline water (IAW) as drinking water. Non-infected control groups and the infected groups treated or not with miltefosine diluted in IAW or TW (IAW+Milt. and TW+Milt.) for 18 weeks.

The weight of the mice was evaluated every three weeks over the four months of the experiment (Fig 2). Until the tenth week, the weight of all animals increased, but in the following weeks, there was a decrease, mainly in the groups treated with miltefosine.

### Weekly follow-up of injury

**Fig 3:**
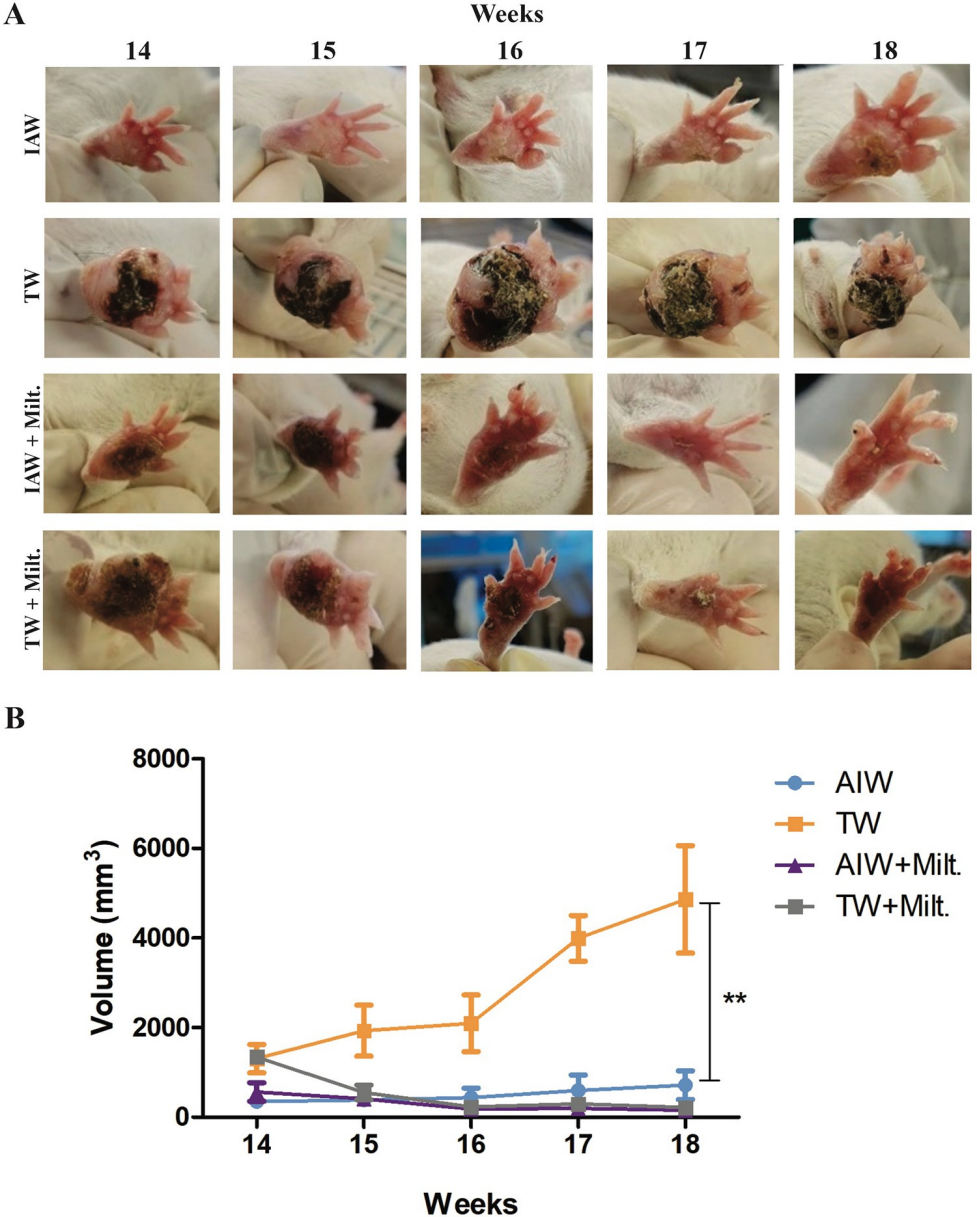
Infected footpads of BALB/c mice. **(A)** Weekly follow-up of groups that received ionized alkaline water (IAW) and tap water (TW) as drinking water, with or without miltefosine treatment for 30 days. **(B)** Lesion volume (mm^3^) of groups. The data are expressed as the mean ± SD (n = 5/group). ∗p ≤0.05, the significant difference between TW and IAW (one-way ANOVA with Tukey test). There was no statistical difference between the groups treated with miltefosine.

A weekly follow-up after infection of the animals (Fig 3A and 3B) was performed to observe the evolution of lesions in the footpads. From week 14, there was an observable difference between the groups that received only TW or IAW. The mice of the TW group had a swollen limb and some necrosis points, different to the IAW group which had slightly swollen paws but had no lesion and no necrosis. At week 17, a necrosis point was observed in most mice that received IAW, which increased in the last week of the experimental period, indicating that the lesion of the IAW group was delayed when compared with the TW group. For the groups treated with miltefosine, initially, the lesion size of the group receiving IAW was smaller than that of the TW group. When miltefosine was administered, the lesion of both groups regressed, but in the last week of treatment, signs of hyperemia could be seen and the volume of the footpads was higher in the TW group compared to the IAW group.

### Blood tests

**Fig 4:**
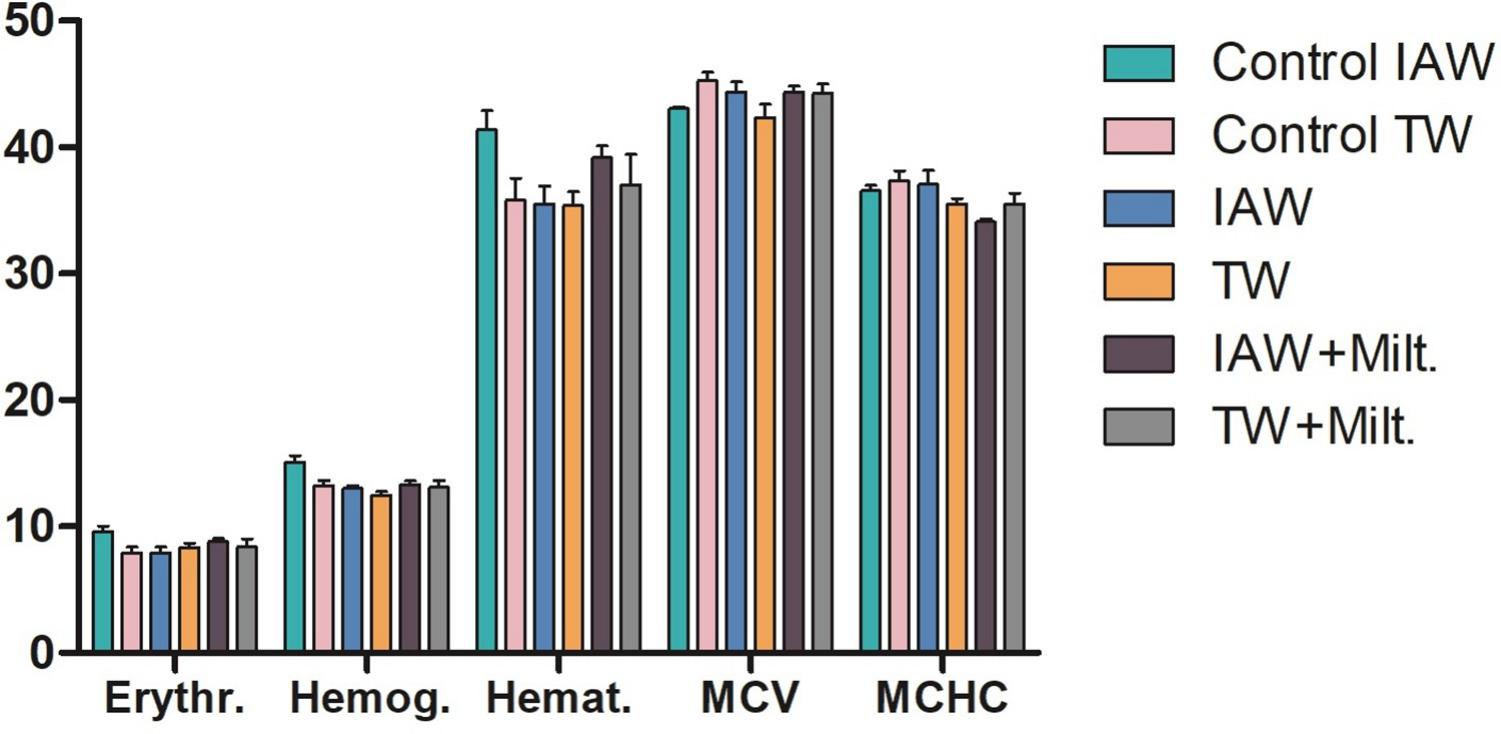
Blood count of BALB/c mice that received either tap water (TW) or ionized alkaline water (IAW) at the end of the experimental period. Erythrocyte (million/μL), hemoglobin (g/dL), hematocrit (%), mean corpuscular volume (MCV;fL), and mean corpuscular hemoglobin concentration (MCHC;%) were analyzed. There was no significant difference between the non-infected control and the infected groups.

**Fig 5:**
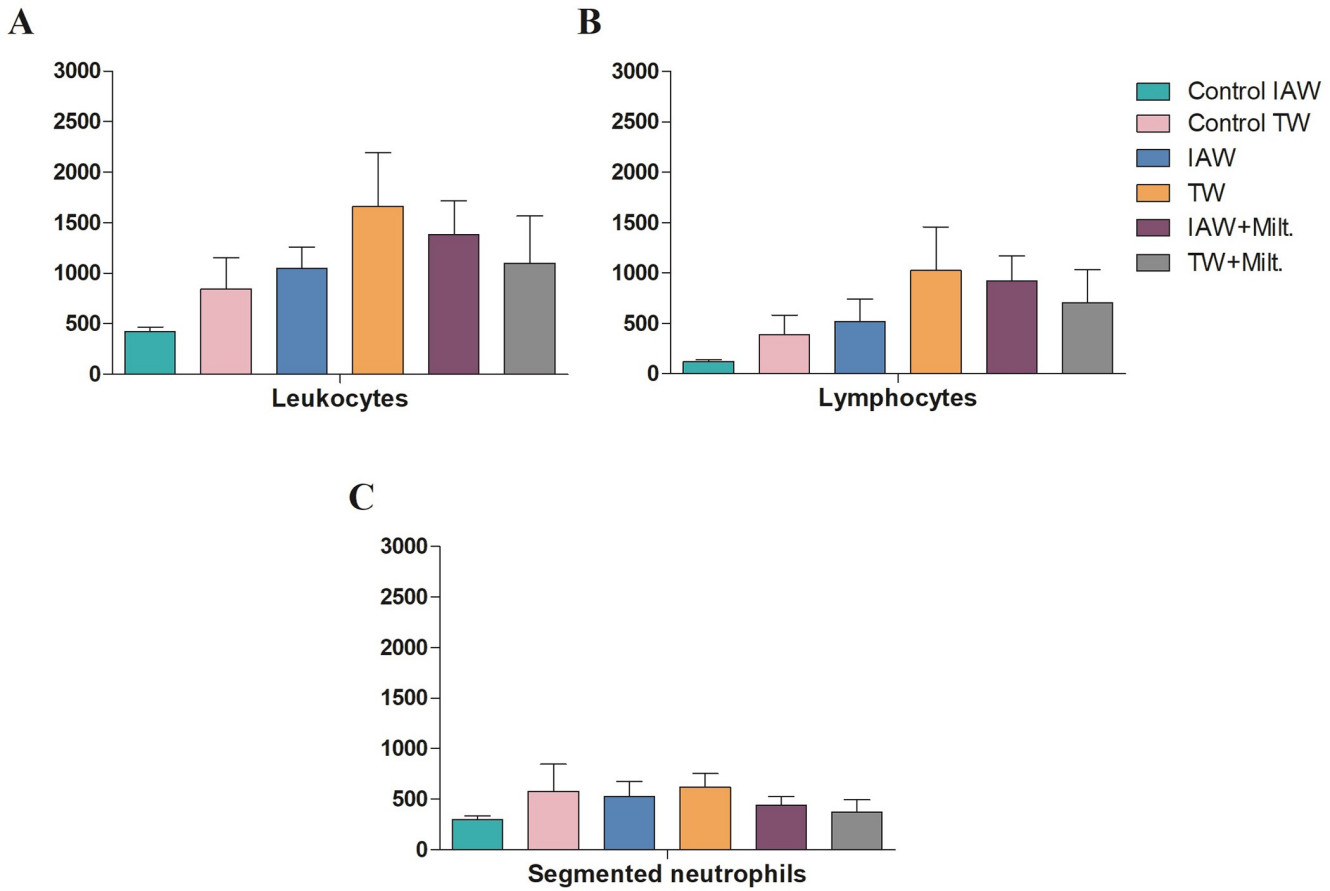
BALB/c leukogram after that received either tap water (TW) or ionized alkaline water (IAW) at the end of the experimental period. **(A)** Leukocytes (count/μL), **(B)** lymphocytes (%), and **(C)** segmented neutrophils (%) were counted. The results showed no significant differences between the control and the infected groups.

To verify whether the IAW affected the hematological parameters and presents signs of toxicity in the mice when compared with TW, a blood count and leukogram with differential count were performed. There were no alterations in the erythrocytes, hemoglobin, hematocrit, mean corpuscular volume (MCV), and mean corpuscular hemoglobin concentration (MCHC) in any group analyzed (Fig 4). On the other hand, slight increases in the number of leukocytes (Fig 5A), lymphocytes (Fig 5B), and segmented neutrophils (Fig 5C) were observed in the non-infected TW group compared with the infected and untreated TW group, but this difference was not significant.

### Biochemical analysis

**Fig 6:**
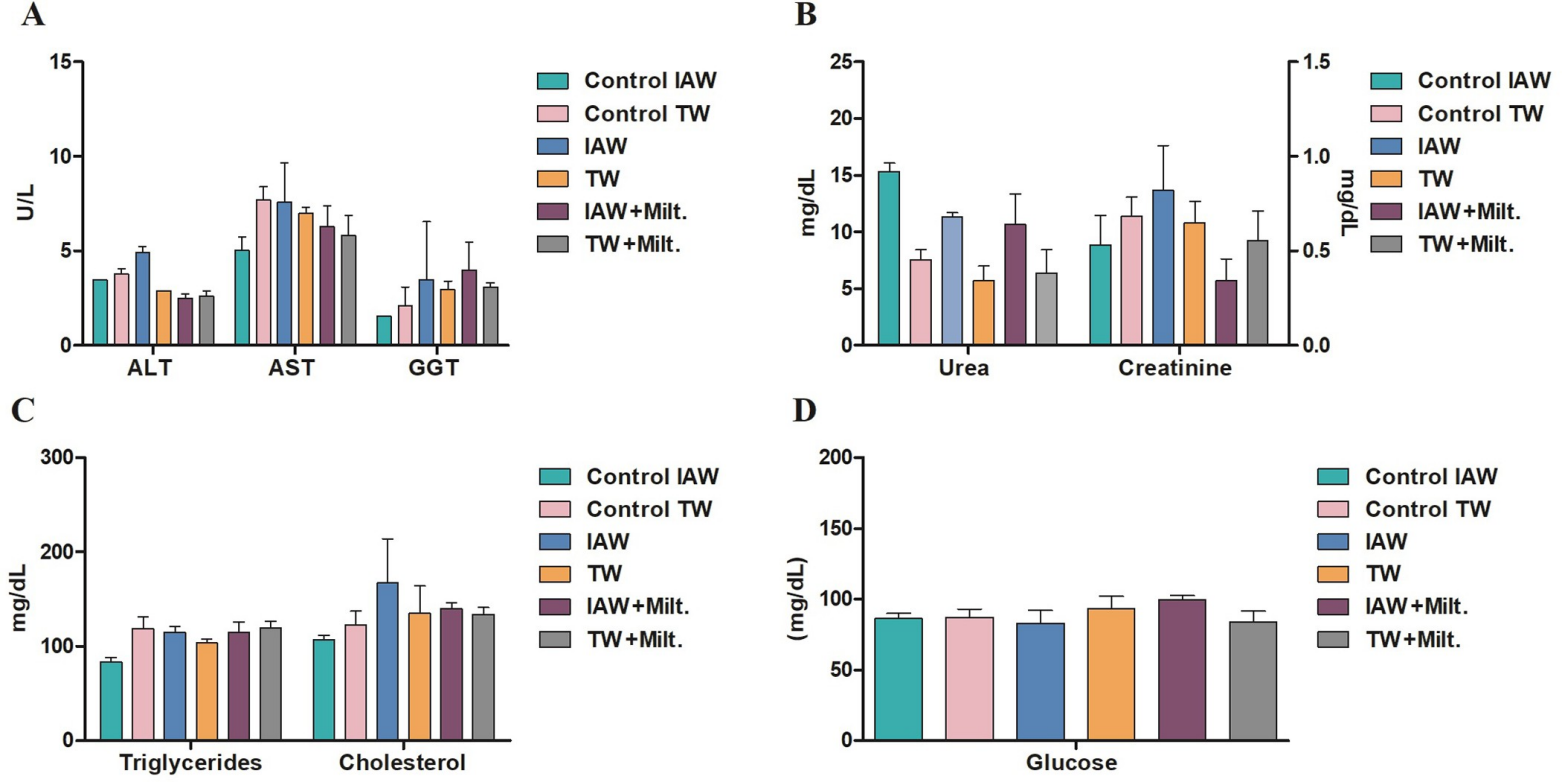
Biochemical analysis of BALB/c mice that received either tap water (TW) or ionized alkaline water (IAW) at the end of the experimental period. **(A)** Alanine aminotransferase (ALT), aspartate aminotransferase (AST) and gamma glutamyl transferase (GGT); **(B)** Urea and creatinine; **(C)** Triglycerides and cholesterol; **(D)** Glucose. There were no statistically significant differences between groups.

For the biochemical analyses, the plasma of the mice was used to evaluate the influence of IAW on liver and renal function, and other important parameters for the diagnosis of metabolic diseases. The liver enzymes, AST, ALT, and GGT of the infected groups receiving IAW and TW showed no statistical difference in relation to the non-infected controls (Fig 6A), neither in the urea and creatinine, which evaluate renal function (Fig 6B). To evaluate the lipid profile of the mice, the triglyceride and cholesterol assays (Fig 6C) were performed, which also showed no significant changes. The glucose test was also performed, in which all infected and non-infected groups remained at similar values (Fig 6D).

## Discussion

Leishmaniasis presents as a wide spectrum of clinical manifestations and is one of the seven most important tropical diseases. Considered a global health problem, this disease is endemic in circumscribed geographical areas in Southern Europe, Northeast Africa, Southeast Mexico, Central and South America, and the Middle East, and is found on all continents except Oceania [17]. The permanence or passage through endemic countries, associated with lack of knowledge and familiarity with leishmaniasis by non-endemic countries leads to delays in the diagnosis and selection of appropriate treatment [18]. Moreover, it is a neglected disease, with a large proportion of the cases registered in underdeveloped countries and, because it mainly affects the low-income population, there are low investments in research and development of new drugs. Therefore, it is necessary to search for new alternatives to improve the treatment of the disease and people’s quality of life [17, 19].

We have demonstrated that the filter effectively removed the chlorine present in the water, since this substance has cytotoxic and genotoxic potential but is widely used for water disinfection [13]. In addition, the filters increased the pH of the water, and that the magnetic field present in the filters can reduced the size of clusters. When water is subjected to a magnetic field an organization of water molecules occurs because of the presence of mineral salts in water [20]; in addition, infrared contributes to the formation of clusters (smaller molecules) [21]. These changes are related to biologically more effective characteristics, such as increased permeation of magnetization by tissues, by smaller groups, which generates greater water diffusion and a water purification and antioxidant effect.

After infection of BALB/c mice with *L*. *amazonensis* promastigotes in the stationary phase of growth, the lesions were forming and gradually evolving over the weeks (Fig 2). Cutaneous leishmaniasis is the most common type of this disease and is characterized in the human disease by lesions [18] that begin with swelling and redness, progressively increasing in size. The lesion typically has a rounded shape, firm consistency, high edges and coarse granulations [19], and in some cases, the progression of the lesion leads to destruction and severe disfigurement of the tissue [22, 23].

Infected mice that received IAW exhibited a significant decrease in the lesion volume compared to mice that received TW, as well as a delay in the development of the macroscopic lesions, such as reduction of swelling and redness. Prior to the start of miltefosine treatment, the animals that drank TW had a bigger lesion than the animals that drank IAW, which indicates that prolonged ingestion of IAW may influence the non-development of lesions. After miltefosine treatment commenced the lesions of both the TW and IAW groups regressed, which was as expected, as it is a medicine of choice for the treatment of cutaneous leishmaniasis [21].

In cutaneous leishmaniasis, as the disease progresses there are structural changes in the epidermal and dermal layers due to the increase of inflammatory cells, which also influences the transepidermal loss of water [22]. An *in vitro* study using electrolyzed water with high pH showed that this water can positively influence the tissue repair process, inducing cell migration. This fact may be due to its antioxidant activity or its ability to reduce the production of intracellular reactive species [23, 24]. In addition, ingestion of IAW can generate physico-chemical changes that are related to biologically more effective characteristics, such as increased permeation of magnetized water by tissues, through smaller clusters, which would lead to a greater elimination of toxins and a depurative effect [20, 25]. When water is subjected to a magnetic field an organization of water molecules occurs because of the presence of mineral salts [20], and infrared contributes to the formation of clusters (smaller molecules) [21]. Purified water also influences the concentration of alkali minerals present in the water source, such as potassium, calcium, and magnesium. Oral administration of a commercially available water alkalizer in mice with melanoma skin cancer was shown to contribute to the control of tumor growth [26]. However, the present study is the first to demonstrate the influence of alkaline water on cutaneous lesions caused by a *Leishmania* sp.

The results of the blood count and leukogram tests were within the normal values for BALB/c mice demonstrating that IAW does not influence these parameters, and thus we can infer that IAW has no toxic effects on blood factors. Likewise, there were no significant differences in biochemical parameters that would indicate an effect on renal and hepatic functions. A study using BALB/c mice infected with *L*. *amazonensis* (1 x 10^7^ cells) in the footpad showed a reduction in the levels of aspartate transaminase and alanine transaminase levels, in addition to the decrease of pro-inflammatory cytokines [27].

Overall, IAW may have positive results for the treatment of cutaneous leishmaniasis due to its characteristics, with the health benefits already demonstrated in both animal and clinical studies [28]. However, there are few studies evidencing the relation between IAW and cutaneous diseases specifically. Health benefits may be related to the ability of IAW to neutralize and eliminate radicals present in cells, preventing oxidative damage to proteins, DNA, and other molecules [11].

This is the first study demonstrating the influence of alkaline water on lesions caused by *Leishmania* sp. Considering the importance of leishmaniasis worldwide, studies proposing alternative and adjuvant therapies are necessary, in addition to broadening the view on the properties of the water that is ingested. The study demonstrated that oral ingestion of ionized alkaline water can contribute to the delayed of the lesion in BALB/c mice infected with *L*. *amazonensis*. Furthermore, the biochemical and hematological parameters remained normal and without significant differences, which suggests that the ingestion of IAW does not cause toxicity to the animals.

## Supporting information

S1 File The ARRIVE guidelines 2.0: Author checklist. (PDF)

S2 File (DOCX)
