# Supplementary material for: Ionized alkaline water reduces injury in BALB/c mice infected with Leishmania amazonensis
Source: PLoS One. 2023 Jul 6;18(7):e0280695. doi: 10.1371/journal.pone.0280695 (PMC10325109; doi:10.1371/journal.pone.0280695)
Supplement: S2 File — (DOCX) [file pone.0280695.s002.docx]

**Supporting Information**

Table 1 - Values of pH and chlorine concentration of tap water (TW) and ionized alkaline water (IAW) used in the experiments.

|  | **Tap water (TW)** | | **Ionized Alkaline Water (IAW)** | |
| --- | --- | --- | --- | --- |
| **Week** | **Chlorine concentration (mg/mL)** | **pH** | **Chlorine concentration (mg/mL)** | **pH** |
| 1 | 1.66 | 6.4 | 0.14 | 8.3 |
|  | 1.67 | 7 | 0.16 | 8.3 |
|  | 1.9 | 6.5 | 0.16 | 8.3 |
| 2 | 1.7 | 6.7 | 0.13 | 7.5 |
|  | 1.8 | 7.2 | 0.21 | 7.5 |
|  | 1.73 | 7.2 | 0.21 | 7.6 |
| 3 | 1.75 | 7.2 | 0.18 | 7.9 |
|  | 1.79 | 6.9 | 0.21 | 7.9 |
|  | 1.95 | 7.2 | 0.21 | 7.9 |
| 4 | 1.6 | 6.8 | 0.14 | 8.3 |
|  | 1.87 | 7.3 | 0.14 | 7.9 |
|  | 1.96 | 6.8 | 0.18 | 8 |
| 5 | 1.8 | 7.3 | 0.18 | 8 |
|  | 1.86 | 6.5 | 0.13 | 8.3 |
|  | 1.52 | 6.7 | 0.15 | 8.1 |
| 6 | 1.86 | 6.8 | 0.13 | 7.9 |
|  | 1.62 | 6.6 | 0.13 | 7.8 |
|  | 1.56 | 6.5 | 0.11 | 7.8 |
| 7 | 1.51 | 6.3 | 0.11 | 8.1 |
|  | 1.85 | 6.7 | 0.11 | 7.9 |
|  | 1.72 | 6.4 | 0.14 | 7.7 |
| 8 | 1.59 | 6.9 | 0.13 | 8 |
|  | 1.68 | 6.8 | 0.15 | 7.7 |
|  | 1.7 | 6.7 | 0.15 | 7.8 |
| 9 | 1.83 | 6.7 | 0.11 | 8 |
|  | 1.6 | 7.1 | 0.14 | 8.1 |
|  | 1.52 | 6.1 | 0.19 | 7.9 |
| 10 | 1.71 | 6.8 | 0.17 | 7.8 |
|  | 1.57 | 6.9 | 0.17 | 7.8 |
|  | 1.64 | 6.9 | 0.15 | 7.7 |
| 11 | 1.46 | 6.8 | 0.14 | 7.7 |
|  | 1.56 | 6.9 | 0.13 | 8 |
|  | 1.61 | 6.5 | 0.15 | 7.8 |
| 12 | 1.68 | 6.8 | 0.12 | 7.8 |
|  | 1.55 | 6.6 | 0.12 | 8 |
|  | 1.63 | 6.8 | 0.14 | 7.8 |
| 13 | 1.46 | 6.5 | 0.13 | 7.9 |
|  | 1.77 | 6 | 0.13 | 7.9 |
|  | 1.89 | 6.4 | 0.15 | 7.9 |
| 14 | 1.75 | 6.6 | 0.13 | 7.8 |
|  | 1.58 | 6.5 | 0.13 | 7.9 |
|  | 1.61 | 6.2 | 0.15 | 8 |
| 15 | 1.66 | 6.8 | 0.13 | 8 |
|  | 1.86 | 6.9 | 0.17 | 8 |
|  | 1.63 | 6.2 | 0.14 | 7.8 |
| 16 | 1.78 | 6.1 | 0.16 | 7.7 |
|  | 1.62 | 6.4 | 0.2 | 7.8 |
|  | 1.7 | 6.5 | 0.18 | 7.8 |
| 17 | 1.63 | 6.6 | 0.2 | 7.8 |
|  | 1.51 | 6.7 | 0.17 | 8 |
|  | 1.68 | 6.7 | 0.2 | 8.3 |
| 18 | 1.67 | 6.5 | 0.18 | 8.3 |
|  | 1.73 | 6.2 | 0.21 | 7.9 |
|  | 1.56 | 6.4 | 0.19 | 8.3 |
| Average | 1.69 | 6.68 | 0.16 | 7.93 |
| SD | 0.13 | 0.31 | 0.03 | 0.20 |

| **Weeks** | **Control IAW** | | | Average | SD | **Control TW** | | | Average | SD | **IAW** | | | Average | SD | **TW** | | | Average | SD | **IAW + Milt.** | | | Average | SD | **TW + Milt.** | | | Average | SD |
| --- | --- | --- | --- | --- | --- | --- | --- | --- | --- | --- | --- | --- | --- | --- | --- | --- | --- | --- | --- | --- | --- | --- | --- | --- | --- | --- | --- | --- | --- | --- |
| **1** | 25.39 | 29.66 | 27.18 | 27.41 | 2.14 | 24.06 | 24.03 | 31.40 | 26.50 | 4.25 | 28.07 | 26.78 | 29.55 | 28.13 | 1.39 | 27.35 | 27.85 | 26.48 | 27.23 | 0.69 | 28.77 | 32.90 | 28.71 | 30.13 | 2.40 | 28.41 | 28.61 | 27.07 | 28.03 | 0.84 |
| **4** | 27.06 | 35.27 | 29.29 | 30.54 | 4.25 | 35.73 | 33.35 | 26.39 | 31.82 | 4.85 | 31.33 | 28.52 | 34.29 | 31.38 | 2.89 | 30.53 | 31.55 | 31.21 | 31.10 | 0.52 | 32.16 | 37.45 | 31.31 | 33.64 | 3.33 | 30.80 | 30.59 | 30.47 | 30.62 | 0.17 |
| **7** | 31.27 | 35.18 | 30.90 | 32.45 | 2.37 | 38.89 | 33.64 | 29.62 | 34.05 | 4.65 | 33.81 | 32.87 | 38.10 | 34.93 | 2.79 | 33.17 | 37.42 | 38.42 | 36.34 | 2.79 | 32.90 | 41.11 | 33.72 | 35.91 | 4.52 | 35.42 | 34.79 | 35.48 | 35.23 | 0.38 |
| **10** | 33.25 | 40.08 | 33.42 | 35.58 | 3.90 | 41.46 | 35.42 | 32.19 | 36.36 | 4.71 | 36.77 | 34.96 | 41.46 | 37.73 | 3.35 | 34.54 | 39.75 | 40.13 | 38.14 | 3.12 | 34.41 | 44.29 | 35.66 | 38.12 | 5.38 | 36.30 | 36.16 | 37.20 | 36.55 | 0.56 |
| **13** | 33.97 | 38.52 | 34.42 | 35.64 | 2.51 | 39.97 | 37.68 | 32.43 | 36.69 | 3.87 | 36.25 | 35.00 | 40.74 | 37.33 | 3.02 | 33.81 | 33.37 | 32.97 | 33.38 | 0.42 | 34.92 | 42.40 | 34.74 | 37.35 | 4.37 | 36.81 | 37.33 | 36.55 | 36.90 | 0.40 |
| **16** | 31.89 | 39.84 | 34.66 | 35.46 | 4.04 | 41.82 | 36.92 | 35.22 | 37.99 | 3.43 | 39.89 | 34.67 | 40.92 | 38.49 | 3.35 | 34.47 | 34.54 | 31.03 | 33.35 | 2.01 | 30.98 | 35.08 | 31.03 | 32.36 | 2.35 | 33.39 | 32.38 | 33.75 | 33.17 | 0.71 |
| **18** | 34.35 | 40.85 | 38.16 | 37.79 | 3.27 | 41.64 | 39.96 | 36.12 | 39.24 | 2.83 | 39.55 | 36.29 | 42.15 | 39.33 | 2.94 | 34.23 | 34.63 | 32.08 | 33.65 | 1.37 | 28.91 | 34.71 | 30.70 | 31.44 | 2.97 | 36.22 | 35.32 | 33.04 | 34.86 | 1.64 |

Fig 2. Weight (g) of non-infected control and infected BALB/c mice. Mouse groups received either tap water (TW) or ionized alkaline water (IAW) as drinking water. Non-infected control groups and the infected groups treated or not with miltefosine diluted in IAW or TW (IAW+Milt. and TW+Milt.) for 18 weeks.

| **Week** | **IAW** | | | | Average | SD | **TW** | | | | Average | SD | **IAW+Milt.** | | | | Average | SD | **TW+Milt.** | | | | Average | SD |
| --- | --- | --- | --- | --- | --- | --- | --- | --- | --- | --- | --- | --- | --- | --- | --- | --- | --- | --- | --- | --- | --- | --- | --- | --- |
| **14** | 320.98 | 389.59 | 286.13 | 393.43 | 347.53 | 52.76 | 1586.68 | 995.31 | 1575.04 | 1072.05 | 1307.27 | 317.50 | 549.39 | 918.05 | 561.74 | 516.94 | 636.53 | 188.63 | * | 1345.89 | 1277.54 | 1415.85 | 1346.43 | 675.58 |
| **15** | 340.99 | 360.82 | 353.77 | 480.99 | 384.14 | 65.08 | 1873.90 | 1591.40 | 2755.80 | 1505.49 | 1931.65 | 571.53 | 268.34 | 525.91 | 432.73 | 529.72 | 439.18 | 122.40 | 334.05 | 727.64 | 561.25 | 588.64 | 552.90 | 163.07 |
| **16** | 293.66 | 473.77 | 260.70 | 719.64 | 436.94 | 210.45 | 2005.09 | 1894.88 | 2989.72 | 1498.28 | 2096.99 | 633.70 | 92.31 | 321.22 | 194.95 | 203.13 | 202.90 | 93.62 | 112.97 | 303.16 | 180.13 | 312.09 | 227.09 | 97.02 |
| **17** | 481.45 | 446.86 | 339.85 | 1110.40 | 594.64 | 349.08 | 3493.09 | 3979.61 | 4511.02 | * | 3994.57 | 2040.09 | 165.63 | 350.33 | 148.42 | 203.95 | 217.08 | 91.81 | 137.36 | 358.45 | 347.30 | 330.70 | 293.45 | 104.68 |
| **18** | 626.99 | 834.13 | 325.10 | 1085.41 | 717.91 | 322.04 | 3964.86 | 4398.83 | 6223.09 | * | 4862.26 | 2620.63 | 135.84 | 231.52 | 157.50 | 109.41 | 158.57 | 52.46 | 127.71 | 323.68 | 281.16 | 129.62 | 215.54 | 101.81 |

Fig 3. Infected footpads of BALB/c mice. **(A)** Weekly follow-up of groups that received ionized alkaline water (IAW) and tap water (TW) as drinking water, with or without miltefosine treatment for 30 days. **(B)** Lesion volume (mm³) of groups. The data are expressed as the mean ± SD (n = 5/group). ∗p ≤0.05, the significant difference between TW and IAW (one-way ANOVA with Tukey test). There was no statistical difference between the groups treated with miltefosine. *Some data points were removed because they were outside the curve and did not represent the mean.

|  | **Control IAW** | | | | Average | SD | **Control TW** | | | | Average | SD | **IAW** | | | | Average | SD | **TW** | | | | Average | SD | **IAW+Milt.** | | | | Average | SD | **TW+Milt.** | | | | Average | SD |
| --- | --- | --- | --- | --- | --- | --- | --- | --- | --- | --- | --- | --- | --- | --- | --- | --- | --- | --- | --- | --- | --- | --- | --- | --- | --- | --- | --- | --- | --- | --- | --- | --- | --- | --- | --- | --- |
| **Erythr.** | 9.35 | 9.77 | 8.21 | 10.10 | 9.36 | 0.82 | 6.97 | 7.21 | 8.49 | 7.67 | 7.59 | 0.67 | 7.11 | 7.54 | 9.19 | 7.97 | 7.95 | 0.90 | 7.97 | 8.38 | 9.33 | 8.76 | 8.61 | 0.58 | 9.04 | 9.25 | 9.38 | 8.73 | 9.10 | 0.28 | 7.36 | 10.10 | 8.62 | 7.52 | 8.40 | 1.26 |
| **Hemog.** | 15.30 | 15.30 | 13.20 | 15.60 | 14.85 | 1.11 | 12.70 | 12.30 | 13.70 | 13.00 | 12.93 | 0.59 | 12.70 | 12.70 | 13.60 | 13.00 | 13.00 | 0.42 | 13.00 | 11.70 | 13.10 | 12.70 | 12.63 | 0.64 | 14.00 | 14.10 | 13.50 | 12.80 | 13.60 | 0.59 | 12.40 | 14.40 | 13.50 | 12.10 | 13.10 | 1.06 |
| **Hemat.** | 41.00 | 42.00 | 36.00 | 43.00 | 40.50 | 3.11 | 33.00 | 32.00 | 39.00 | 34.00 | 34.50 | 3.11 | 32.00 | 35.00 | 39.00 | 36.00 | 35.50 | 2.89 | 36.00 | 34.00 | 38.00 | 37.00 | 36.25 | 1.71 | 41.00 | 41.00 | 40.00 | 38.00 | 40.00 | 1.41 | 33.00 | 43.00 | 39.00 | 33.00 | 37.00 | 4.90 |
| **MCV** | 43.30 | 43.10 | 43.40 | 42.80 | 43.15 | 0.26 | 46.80 | 44.00 | 45.60 | 46.60 | 45.75 | 1.28 | 44.30 | 46.20 | 42.40 | 44.70 | 44.40 | 1.56 | 44.70 | 40.00 | 40.20 | 42.00 | 41.73 | 2.18 | 45.70 | 44.20 | 42.50 | 43.40 | 43.95 | 1.36 | 45.00 | 42.10 | 45.60 | 44.30 | 44.25 | 1.53 |
| **MCHC** | 37.80 | 36.30 | 37.10 | 36.10 | 36.83 | 0.78 | 39.00 | 38.80 | 35.40 | 38.00 | 37.80 | 1.66 | 40.30 | 36.50 | 34.90 | 36.50 | 37.05 | 2.29 | 36.50 | 34.90 | 34.90 | 34.50 | 35.20 | 0.89 | 33.90 | 34.50 | 33.80 | 33.80 | 34.00 | 0.34 | 37.50 | 33.90 | 34.40 | 36.30 | 35.53 | 1.67 |

Fig 4. Blood count of BALB/c mice that received either tap water (TW) or ionized alkaline water (IAW) at the end of the experimental period. Erythrocyte (million/µL), hemoglobin (g/dL), hematocrit (%), mean corpuscular volume (MCV;fL), and mean corpuscular hemoglobin concentration (MCHC;%) were analyzed. There was no significant difference between the non-infected control and the infected groups.­

| **A** | **Control IAW** | | | | | Average | SD | **Control TW** | | | | | Average | SD | **IAW** | | | | Average | SD | **TW** | | | | | Average | SD | **IAW+Milt.** | | | | | Average | SD | **TW+Milt.** | | | | Average | SD |
| --- | --- | --- | --- | --- | --- | --- | --- | --- | --- | --- | --- | --- | --- | --- | --- | --- | --- | --- | --- | --- | --- | --- | --- | --- | --- | --- | --- | --- | --- | --- | --- | --- | --- | --- | --- | --- | --- | --- | --- | --- |
| Leukocytes | 400 | 600 | 300 | 400 | 400 | 420.00 | 97.98 | 200 | 500 | 1700 | 300 | 1500 | 840.00 | 631.19 | 800 | 1500 | 1300 | 600 | 1050.00 | 364.01 | 600 | 1100 | 3000 | 2900 | 700 | 1660.00 | 1066.96 | 400 | 2700 | 1700 | 700 | 1200 | 1340.00 | 811.42 | 300 | 2400 | 600 | 1100 | 1100.00 | 803.12 |

| **B** | **Control IAW** | | | | | Average | SD | **Control TW** | | | | | Average | SD | **IAW** | | | | Average | SD | **TW** | | | | | Average | SD | **IAW+Milt.** | | | | | Average | SD | **TW+Milt.** | | | | Average | SD |
| --- | --- | --- | --- | --- | --- | --- | --- | --- | --- | --- | --- | --- | --- | --- | --- | --- | --- | --- | --- | --- | --- | --- | --- | --- | --- | --- | --- | --- | --- | --- | --- | --- | --- | --- | --- | --- | --- | --- | --- | --- |
| Lymphocytes | 88 | 174 | 138 | 116 | 108 | 124.80 | 29.36 | 235 | 272 | 90 | 960 | * | 389.25 | 336.47 | 184 | 615 | 1118 | 162 | 519.75 | 389.77 | 162 | 704 | 1800 | 2262 | 217 | 1029.00 | 852.22 | 992 | 180 | 1782 | 816 | 322 | 818.40 | 567.91 | 129 | 1584 | 264 | 836 | 703.25 | 573.60 |

| **C** | **Control IAW** | | | | | Average | SD | **Control TW** | | | | | Average | SD | **IAW** | | | | Average | SD | **TW** | | | | | Average | SD | **IAW+Milt.** | | | | | Average | SD | **TW+Milt.** | | | | Average | SD |
| --- | --- | --- | --- | --- | --- | --- | --- | --- | --- | --- | --- | --- | --- | --- | --- | --- | --- | --- | --- | --- | --- | --- | --- | --- | --- | --- | --- | --- | --- | --- | --- | --- | --- | --- | --- | --- | --- | --- | --- | --- |
| Segmented neutrophils | 312 | 426 | 162 | 284 | 292 | 295.20 | 83.99 | 265 | 1360 | 210 | 480 | * | 578.75 | 462.20 | 616 | 885 | 182 | 438 | 530.25 | 256.41 | 438 | 396 | 1140 | 638 | 483 | 619.00 | 273.05 | 608 | 220 | 783 | 384 | 272 | 453.40 | 212.01 | 171 | 720 | 336 | 264 | 372.75 | 208.84 |

Fig 5. BALB/c leukogram after that received either tap water (TW) or ionized alkaline water (IAW) at the end of the experimental period. **(A)** Leukocytes (count/µL), **(B)** lymphocytes (%), and **(C)** segmented neutrophils (%) were counted. The results showed no significant differences between the control and the infected groups. *Some data points were removed because they were outside the curve and did not represent the mean.

| **A** | **Control IAW** | | | Average | SD | **Control TW** | | | Average | SD | **IAW** | | | Average | SD | **TW** | | | Average | SD | **IAW+Milt.** | | | Average | SD | **TW+Milt.** | | | Average | SD |
| --- | --- | --- | --- | --- | --- | --- | --- | --- | --- | --- | --- | --- | --- | --- | --- | --- | --- | --- | --- | --- | --- | --- | --- | --- | --- | --- | --- | --- | --- | --- |
| ALT (U/L) | 3.30 | 3.49 | 3.57 | 3.45 | 0.14 | 3.49 | 3.97 | 4.07 | 3.84 | 0.31 | 4.66 | 5.03 | 5.24 | 4.98 | 0.30 | 2.91 | 2.90 | 2.91 | 2.91 | 0.01 | 2.91 | 2.33 | 2.33 | 2.52 | 0.34 | 2.33 | 2.79 | 2.91 | 2.68 | 0.31 |
| AST (U/L) | 6.40 | 4.66 | 4.07 | 5.04 | 1.21 | 5.82 | 7.57 | 8.73 | 7.37 | 1.46 | 3.49 | 8.73 | 10.48 | 7.57 | 3.63 | 6.98 | 7.57 | 6.40 | 6.98 | 0.58 | 4.07 | 5.24 | 10.48 | 6.60 | 3.41 | 6.98 | 3.49 | 8.15 | 6.21 | 2.42 |
| GGT (U/L) | 1.54 | 1.48 | 1.61 | 1.54 | 0.07 | 3.09 | 1.16 | 1.24 | 1.83 | 1.09 | 6.56 | 0.39 | * | 3.47 | 3.68 | 3.86 | 2.32 | 2.70 | 2.96 | 0.80 | 6.95 | 2.32 | 2.70 | 3.99 | 2.57 | 3.47 | 2.70 | 3.09 | 3.09 | 0.39 |

| **B** | **Control IAW** | | | Average | SD | **Control TW** | | | Average | SD | **IAW** | | | Average | SD | **TW** | | | | Average | SD | **IAW+Milt.** | | | | Average | SD | **TW+Milt.** | | | | Average | SD |
| --- | --- | --- | --- | --- | --- | --- | --- | --- | --- | --- | --- | --- | --- | --- | --- | --- | --- | --- | --- | --- | --- | --- | --- | --- | --- | --- | --- | --- | --- | --- | --- | --- | --- |
| Urea (mg/dL) | 14.64 | 16.10 | 14.46 | 15.07 | 0.90 | 8.78 | 8.05 | 5.86 | 7.56 | 1.52 | 10.89 | 11.71 | 10.98 | 11.20 | 0.45 | 9.52 | 2.93 | 8.05 | 4.39 | 5.12 | 2.64 | 5.12 | 19.03 | 14.64 | 8.78 | 14.15 | 5.14 | 3.66 | 2.20 | 10.25 | 9.52 | 7.32 | 4.45 |
| Creatinine (mg/dL) | 0.63 | 0.74 | 0.23 | 0.53 | 0.27 | 0.69 | 0.86 | 0.51 | 0.69 | 0.17 | 1.09 | 0.34 | 1.03 | 0.82 | 0.41 | 0.86 | 0.63 | 0.46 | * | 0.54 | 0.32 | 0.11 | 0.74 | 0.17 | 0.46 | 0.46 | 0.29 | 0.46 | 0.23 | 0.97 | 0.57 | 0.59 | 0.37 |

| **c** | **Control IAW** | | | | Average | SD | **Control TW** | | | | Average | SD | **IAW** | | | | Average | SD | **TW** | | | | Average | SD | **IAW+Milt.** | | | | Average | SD | **TW+Milt.** | | | | Average | SD |
| --- | --- | --- | --- | --- | --- | --- | --- | --- | --- | --- | --- | --- | --- | --- | --- | --- | --- | --- | --- | --- | --- | --- | --- | --- | --- | --- | --- | --- | --- | --- | --- | --- | --- | --- | --- | --- |
| Triglycerides (mg/dL) | 72.26 | 89.34 | 97.08 | 84.72 | 85.85 | 9.00 | 82.64 | 150.75 | 139.25 | 115.09 | 135.03 | 18.20 | 107.08 | 113.87 | 104.06 | 133.49 | 114.62 | 11.46 | 115.66 | 89.81 | 105.09 | 103.11 | 103.42 | 9.19 | 90.85 | 155.00 | 107.92 | 108.02 | 115.45 | 23.88 | 135.85 | 108.87 | 128.30 | 105.19 | 119.55 | 12.87 |
| Cholesterol (mg/dL) | 116.05 | 93.49 | 97.74 | 109.57 | 104.21 | 9.02 | 108.39 | 166.17 | 147.02 | 94.95 | 136.05 | 36.85 | 100.52 | 305.67 | 142.99 | 117.93 | 166.78 | 81.60 | 39.96 | 220.47 | 117.79 | 145.42 | 130.91 | 64.57 | 148.49 | 154.82 | 129.48 | 123.91 | 139.18 | 12.83 | 152.25 | 134.28 | 133.73 | 114.65 | 133.73 | 13.29 |

| **D** | **Control IAW** | | | | Average | SD | **Control TW** | | | | Average | SD | **IAW** | | | | Average | SD | **TW** | | | | Average | SD | **IAW+Milt.** | | | | Average | SD | **TW+Milt.** | | | | Average | SD |
| --- | --- | --- | --- | --- | --- | --- | --- | --- | --- | --- | --- | --- | --- | --- | --- | --- | --- | --- | --- | --- | --- | --- | --- | --- | --- | --- | --- | --- | --- | --- | --- | --- | --- | --- | --- | --- |
| Glucose (mg/dL) | 75.49 | 93.17 | 96.16 | 81.04 | 86.46 | 8.50 | 77.97 | 81.55 | 109.52 | 84.76 | 88.45 | 12.40 | 85.65 | 77.33 | 62.13 | 106.23 | 82.84 | 15.93 | 116.40 | 75.36 | 91.03 | 73.53 | 89.08 | 17.18 | 102.60 | 94.24 | 102.52 | 94.27 | 98.41 | 4.15 | 99.96 | 94.36 | 71.61 | 69.64 | 83.89 | 13.43 |

Fig 6. Biochemical analysis of BALB/c mice that received either tap water (TW) or ionized alkaline water (IAW) at the end of the experimental period. **(A)** Alanine aminotransferase (ALT) and aspartate aminotransferase (AST); **(B)**. gamma glutamyl transferase (GGT); **(C)** urea; **(D)** creatinine; **(E)** glucose; **(F)** triglycerides; and **(G)** cholesterol. There were no statistically significant differences between groups. *Some data points were removed because they were outside the curve and did not represent the mean.
